# Indents

**Published:** 1920-08

**Authors:** 


### INDENTS

**☞ Brief Articles of Technical or Scientific Value will receive notice and comment. Contributions invited.**

---

Dignity of Dental Service. When the time comes that dentists think more of the money they make than of the service they render; when they expend their energy in exploiting the people rather than conserving their welfare; when they exalt the God of Mammon above the Divinity of Service; when the sordid and selfish shall assume the ascendency over the sublimity of sacrifice, then shall this profession of ours sink into the nethermost depths of degradation, and we shall be known as men not worthy of professional status, not fit to associate with those of culture and refinement, not even entitled to a place beside the common day laborer, because we have besmirched our calling and betrayed a trust reposed in us by the sturdy old pioneers of the days gone by, who, by their devotion and self-sacrifice, have handed down to us a profession of high ideals, whereby we were enjoined to serve humanity in an honorable way, rather than to use our calling as a cloak to impose upon the people.

It remains for you and for me to choose whether we shall exalt commercialism above professionalism, or whether we as professional practitioners shall so conduct our affairs that, while reaping a legitimate material reward, we may be able to look every man in the face and not hang our heads in shame.—C. N. Johnson, in *Good Health.*

---

Dental Cysts. A differential diagnosis between the chronic inflammatory reaction in the peridental membrane and surrounding osseous tissue commonly referred to as a dental granuloma and dental or root cysts, is one among many other frequently neglected phases of dental and oral pathology. They are alike in only one respect, viz., that they are reactions to degrees of irritation, with the exception that while the root cyst may be the manifestation of an irritation other than bacterial, the granuloma or chronic dento-alveolar abscess is invariably the reaction to bacterial infection. From the standpoint of pathologic histology, the difference between a root cyst and a so-called dental granuloma is striking and unequivocal. The dental granuloma is the creation to a chronic low-grade infection. It is characterized by the presence, within the enveloping fibrous sac, of inflammatory cells in large numbers called thither for certain specific purposes. The dental or root cyst, provided it has not been invaded by bacteria, is free from inflammatory cells, and is made up of an external fibrous connective tissue sac around a wall of less dense connective tissue fibers and cells and one or more layers of epithelial cells forming the lining of the cyst cavity. The dental or root cyst, again providing that it has not become infected subsequent to its formation, does not give rise in the surrounding osseous tissue to a chronic osteomyelitis as is invariably the case with a chronic dento-alveolar abscess; the lesion in the bone is one of absorption and deposition, the absorption taking place more rapidly than the deposition, eventually producing a bony wall so thin as to bulge out over the cyst, producing upon interrupted pressure a ''parchment crackling'' sensation, or in extreme cases complete absorption of the wall will occur, bringing the cyst in contact with the mucous membrane of the mouth.

In the diagnosis of dental cysts the radiograph is of considerable value as the outline of a cyst is usually very definite, establishing a clear line of demarcation between the diseased and surrounding normal areas. These cysts may develop in the maxilla or in the mandible without reference to any special location. The early stages of cyst development are unrecognizable, and radiographically they can be detected only after they have attained a certain size and therefore have induced a loss of a certain area of osseous substance. Pain is not an accompanying symptom of dental cysts, but when present is due to the pressure of the cyst upon nerve filaments. There is no particular discomfort other than that due to the increasing size of the cyst which is in most cases the reason which leads patients to seek relief, unless it be that through unnecessary manipulations through the root canal the cyst has been perforated and infected and symptoms of an acute or sub-acute process are present.

Once a diagnosis of dental cyst has been made, its treatment should be undertaken along surgical lines. It can not be reached by the procedures, mechanical and therapeutic, usually employed in the treatment of infected root canals giving rise to granulomata. The etiology and pathologic anatomy of all forms of cysts of the jaws, including these simple dental or root cysts, have much in common with the etiology and pathologic anatomy of true neoplasms. There is as much therapeutic logic in attempting to bring about the disappearance of a dental cyst by means of root canal medication as there would be in endeavoring to eradicate an epithelioma by means of drugs. —*The Pacific Dental Gazette.*

---

An Aid for Contour Clasps. Take an individual impression in "Complaster," dry out thoroughly, and pour up with Melotte's metal. This model can be swaged upon or hammered against in swaging the clasp without the fear of breaking usually occurring with an amalgam or cement tooth die.—L. M. Baughman.

---

Printing on Radiographs. Finding it impossible to secure small lead letters for printing on X-ray negatives by laying them on the negative during exposure, I made my own as follows: A sheet of pink wax was warmed slightly and slipped into a typewriter. Then with the ribbon slipped to one side, the desired lettering was stamped in the wax. Removing the wax, the letters were filled with a soft amalgam and allowed to set. The excess amalgam was cleaned off while soft. The wax was trimmed to leave a narrow margin about the lettering, then covered with the adhesive paper such as used in stores in place of string, or with adhesive of any sort, on which the lettering is again written in ink in order that one may know which lettering he is using. If carefully done, clear, sharp printing will result.—John Waters, D.D.S.

---

ROOT CANAL THERAPEUTICS. There has been much neglect and indifference to root canal operations in past years, and even today there is evidence of a shirking of responsibility in this work. We must face the situation squarely and learn to make a proper diagnosis of conditions, and extract such teeth as can not safely be retained, and by proper treatment correct such conditions as can be corrected, and lastly and most important of all, see to it that *the fillings we place in root canals today are as near perfect technically as it is possible for us to make them.*—E. D. Cooledge.

---

PERFORATION FROM DRILL CANAL. If the perforation is of recent date, use a soothing antiseptic and over the opening place a layer of tin foil, against which insert amalgam.—*Oral Health.*

---

SENSITIVENESS AT THE NECKS OF THE TEETH. A saturated solution of carbonate of potassium in glycerine, applied to the points of sensitiveness, will counteract the action of ferments and allay hyper-sensitiveness.—A. C. H.

---

REMOVAL OF DEVITALIZED PULPS. To harden the contents of root canals and facilitate their removal after disintegration with arsenic, saturate a piece of cotton or punk with a five per cent. solution of formalin and place over the stump after removal of the bulbous portion of the pulp, being careful in multi-rooted teeth to have the cotton large enough to cover all of the stumps. Over the cotton place a Teague disk and place temporary stopping or cement over the disk. Three days will effect complete devitalization and hardening.—*Oral Health.*

---

Temporary Crowns. One often finds it difficult to restore, temporarily, anterior teeth, especially uppers, during devitalization, treatment or preparation of the root (and crown) for a Richmond crown. When the tooth is badly broken down, a celluloid synthetic porcelain form, fitted to the root and clearing occlusion may be filled with cement, and while the cement is soft, place it over the broken-down crown. In this way, a devitalizing agent may be sealed in as well as restoring temporarily the natural crown of the tooth. If the root has been prepared for a crown, trim the synthetic crown form to fit the root, going under the gum slightly and clearing occlusion. Select a base metal crown post and insert into the canal. Fill with white temporary stopping, warming and packing with a burnisher. Force this crown over the root. Allow to cool and remove with post attached. Place a little warm temporary stopping in the root and press the crown to place. This crown may be removed and used more than once. It resembles a tooth in which the nerve has been devitalized and does not look unsightly.—Harold W. Hoag, D.D.S., in *Oral Health.*

---

Practical and Scientific Casting. The practice of every art implies a certain knowledge of natural causes and effects; and the improvement of the art depends upon our learning more and more of the properties and powers of natural objects, and discovering how to turn the properties and powers of things, and the connections of cause and effect among them to our own advantage. The most practical thing in the world is the most scientific. In fact, nothing becomes really practical until it is put on a scientific basis; that is, until its relation to other facts is known and noted. To be scientific as well as practical a cast clasp should not be a mere inert, bulky ring of metal, but should be resilient to a slight degree; not much heavier than a wrought clasp of 24 gauge (B and S Standard), but most important of all, should grasp the tooth tighter at its ends than at anywhere else. This last-named property is obtained by forming under tension a casting wax pattern on a metal tooth. Owing to the shrinkage of the casting wax used (Taggart's), this tension is released when the pattern is chilled and removed from the metal tooth model, causing the two ends of the pattern to draw together slightly. This, with a further contraction of the casting metal, cast in a cool mould, causes the finished clasp to grasp tighter at its ends than at any other point in its circumference. This griping is highly desirable.—*Oral Health.*

---

WELDING AMALGAM. Sometimes a small part of an amalgam restoration is broken off in spite of the care of the patient and care by the operator. Such damage may be permanently repaired without removing the entire restoration by the following method:

Make a mixture of amalgam in the usual manner, then squeeze out the surplus mercury, but do not throw it away, for it contains tin. Take this mercury and tin and burnish it on the surface that you wish to add to and then build out with the amalgam. The rubber dam is not needed, for this weld can be made in the presence of moisture.—*F. W. F.*

